# Self-Evaluation Differences Among Swedish Children and Adolescents Over a 30-Year Period

**DOI:** 10.3389/fpsyg.2020.00802

**Published:** 2020-04-28

**Authors:** Eva V. Hoff, Daiva Daukantaitė, Pirjo Birgerstam

**Affiliations:** Department of Psychology, Lund University, Lund, Sweden

**Keywords:** self-concept, self-evaluation, children, adolescents, cohort differences, generational differences, gender differences

## Abstract

International research has found changes in how today’s young people evaluate themselves. The present Swedish research contributes with new findings by distinguishing different patterns of change in self-evaluation in two age groups. The study investigates generational and gender differences in five self-evaluation dimensions in two samples, one from 1983 (*N* = 3052 10–16-year-old students) and one from 2013 (*N* = 1303 10–18-year-old students). Three age groups were analyzed. The *generational comparison* for primary school (*ages 10–12*) showed higher scores in 2013 than in 1983 for all five self-evaluation dimensions. Interactions between generation and gender were found for psychological well-being, relations to others, school competence evaluations, and the total score, demonstrating, in contrast to international research, a greater increase for girls than for boys. Noteworthy is that girls in primary school had higher scores in 2013. *The generational comparison* for *lower secondary school* (*ages 13–15*) demonstrated higher scores for school competence, relational self-evaluations, and a total higher score in 2013. Interactions between generation and gender were found for total, physical, and psychological well-being evaluation scores, indicating an increase for boys and a decrease for girls in 2013 compared to 1983. *The gender comparison* for secondary *school* (*ages 16–18, 2013*), showed gender differences for physical, psychological well-being, school competence evaluations, and for the total score to the advantage of boys. The study discusses changes in self-evaluation in relation to phenomena such as permissive child-rearing, decreased demands in school, increased self-enhancement behavior through social media, and narrow body ideals in today’s society. The study recommends that interventions directed toward groups with low self-evaluation scores should be considered.

## Introduction

International studies suggest self-evaluation differences between today’s young generation, ‘the Millennials’^1^ and those born some decades earlier, ‘Generation X’^2^ (Twenge and Campbell, 2001; Gentile et al., 2010; Bachman et al., 2011; Von Soest and Wichstrøm, 2014). Some scholars maintain that there is an increased self-focus in today’s society owing to changes in cultural values and ideas (Twenge and Campbell, 2009). Simultaneously, scientific reports in many Western countries show a decrease in well-being and an increase in depression and self-harm behavior among adolescents (Collishaw et al., 2010; Fleming et al., 2014). Phenomena, such as an increase in permissive child-rearing methods, changed demands in school, increased self-enhancement behavior through the Internet and social media, and increasingly narrow body ideals, are used to explain why Millennials demonstrate seemingly contradictory trends – increases in self-esteem and decreases in subjective well-being. Eckersley and Dear (2002; also Park et al., 2014) claim that these results do not conflict because the increase in self-focus in individualistic cultures produces inflated feelings of self-worth. These inflated feelings of self-worth are accompanied by unrealistic expectations on choice, opportunity, and attainment in many aspects of life. Because such high expectations cannot be met, they may lead to experiences of failure that result in decreased subjective well-being (Eckersley and Dear, 2002; Park et al., 2014).

Scientific studies (Hagquist, 2010; Petersen et al., 2010; National Board of Health and Welfare, 2013) and the media in Sweden (Glimberg, 2012; Sjöberg, 2013) report that depression and anxiety have increased among adolescents, but there is a lack of research on how self-evaluations in Sweden have changed from Millennials to Generation X. Therefore, the present study investigates self-evaluation differences between two cross-sectional samples from 1983 and 2013.

There are several self-constructs, of which one is *dimensional self-evaluation* (also termed self-concept), that refer to how individuals evaluate different self-aspects, such as physical self-image, psychological self-image, family relations, friend relations, and school competencies. Another self-construct is *global self-esteem*, which refers to how much value individuals generally attribute to themselves (Harter, 2012; Orth et al., 2014b) measured with items such as *I am satisfied with myself*. This is the construct used most often in studies of generational self-trends. Meta-studies have primarily found increases in global self-esteem (Twenge and Campbell, 2001; Gentile et al., 2010; Von Soest and Wichstrøm, 2014). However, researchers have also demonstrated small or non-existent changes between generations in global self-esteem (Trzesniewski et al., 2008; Trzesniewski and Donnellan, 2010; Orth and Robins, 2014; Orth et al., 2014a). The construct global self-esteem has been criticized. One influential review (Baumeister et al., 2003) has demonstrated that global self-esteem poorly predicts academic achievement, work performance, and health-related behaviors. This is one reason to use other self-constructs in generational comparisons, such as *dimensional self-evaluations*, which in contrast to global self-esteem, are consistently associated with performance (within the same dimension). For instance, academic self-evaluation predicts academic performance well (Valentine et al., 2004; Marsh et al., 2006). There is a lack of studies in generational comparisons of dimensional self-evaluations, particularly in the Nordic countries. In the present study, we examine whether two cross-sectional samples of Swedish students (from 1983 and 2013) score differently on five self-evaluation dimensions. As there are developmental differences between primary and secondary school students, the groups’ self-evaluation scores have been analyzed separately in research.

### Generational Differences in Self-Measures in Different Age Groups

In American studies, larger cohort differences have been found among children than among adolescents. The Gentile et al. (2010) meta-analysis on global self-esteem change between 1988 and 2006 reported a large difference (Cohen’s *d* = 0.78) in the age group 11–13. Self-esteem was higher in 2006. Gentile et al. (2010) have demonstrated smaller differences (*d* = 0.39 for the whole sample and *d* = 0.17 for Caucasian participants) for secondary school students (ages 14–17). The Twenge and Campbell (2010) meta-analysis of 14-year-olds’ global self-esteem found a very small difference (*d* = 0.12) between the years 1991 to 2007. Studies of generational differences in dimensional self-evaluations are few.

Very few generational comparisons have been undertaken in the Nordic countries on global self-esteem or on different self-evaluation dimensions. The few comparisons that do exist only compare one age group (Wångby et al., 2005; Frisén and Anneheden, 2014), include a limited time period (Frisén and Anneheden) or only study one dimension or solely global self-esteem (Wångby et al., 2005; Frisén et al., 2014). Frisén and Anneheden did not detect any changes between 2000 and 2010 in Swedish 10-year-olds’ body-related self-evaluation. A comparison of 15-year-old girls from two Swedish samples (1970 and 1996) revealed a tendency toward more global self-esteem problems in 1996 than in 1970 (Wångby et al., 2005). A Norwegian study compared 16-year-old adolescents’ global self-esteem and also the dimension physical appearance between the years 1992 and 2010 (Von Soest and Wichstrøm, 2014). They found a small increase in trends in both the appearance dimension (*d* = 0.19 for girls and 0.27 for boys) and the global self-esteem dimension (*d* = 0.12 for girls and 0.19 for boys). Our study investigates self-evaluation differences in two different age groups (10–12 and 13–15 years) in order to present separate generational trend analyses for primary and for lower secondary school. Gender differences is another self-related aspect that influences self-evaluation trends in different stages of development.

### The Development of Gender Differences in Self-Measures

Most gender studies of global self-esteem find gender differences to the advantage of males. The effect sizes of the self-esteem gender differences over a life span, however, are generally small (*d* = 0.25 in Bleidorn et al.’s, 2016; *d* = 0.21 in Kling et al., 1999). The largest gender differences have been found during adolescence (*d* = 0.33 in Kling et al., 1999 for 15–18-year-olds).

Girls have been found to score equally with boys on global self-esteem in the early years (4–9 years in Noordstar et al., 2016; 9–12 years in Robins et al., 2002). Girls score even higher than boys in self-evaluations in some dimensions, such as reading (ages 7–11 in Marsh, 1989), honesty (at the ages of 10 and 12), and school competence and peer relations (at the age of 12: Orth et al., 2014b).

Both girls and boys show a drop in global self-esteem worldwide around the transition to lower secondary school at the age of 12 or 13 (Marsh, 1989; Twenge and Campbell, 2001; Robins et al., 2002; Cantin and Boivin, 2004). This decrease in self-esteem runs parallel to an increase in cognitive ability to evaluate oneself more realistically and in relation to others (Harter, 2012). Global self-esteem increases from the age of 15 or 16 (Erol and Orth, 2011; Bleidorn et al.’s, 2016). A widening of the gender gap begins around the transition to lower secondary school as indicated in some Western countries (Marsh, 1989 in Australia; Twenge and Campbell, 2001 in the United States) in that girls’ self-esteem declines more than that of boys. Girls’ self-esteem does not recover from the adolescent dip until the last year of secondary school and during college years (at the age of 17 or 18). One Swedish study has indicated that the turning point for young men might come somewhat later, around the age of 21 (Frisén et al., 2014). Young men’s scores on global self-esteem (Robins et al., 2002), the five-dimensional self-evaluation (Räty et al., 2005), and the body-related self-dimension (Frisén et al., 2015) were found to decrease slightly during late adolescence. Women do not reach the same level of global self-esteem as men until very old age (age 80), despite that the gender gap narrows, as shown in two studies (US sample: Orth et al., 2010; mixed sample: Robins et al., 2002). However, no significant gender difference in global self-esteem has been found by the age of 31 in a Norwegian sample (13–31 years of age) (von Soest et al., 2016). Cross-cultural variation has been found in gender differences (see Bleidorn et al.’s, 2016 comparison of 48 countries).

Larger gender differences have been found in dimensional rather than in global self-measures in secondary school (ages 11–17) (Twenge and Campbell, 2001; Gentile et al., 2009). The dimension-specific gender differences that have been highlighted show that boys score higher on physical self-evaluation (Feingold and Mazella, 1998; von Soest et al., 2016; Gentile et al., 2009) and math self-evaluation (Marsh, 1989), whereas girls score higher on verbal and spiritual self-evaluation (Marsh, 1989), close friendships (von Soest et al., 2016), and for behavioral conduct self-evaluation (Gentile et al., 2009).

Some inferred explanations for the gender gap in most self-dimensions are: *Stereotypical gender roles* that are enforced in segregated play groups during the early school years. High global self-esteem in adolescence is described as a gender role violation for girls (Kling et al., 1999). *The cultural focus on women’s physical appearance* is another suggested cause and is manifested in all types of Western media (Hargreaves and Tiggemann, 2004; Rodgers et al., 2015). Although appearance is related to self-evaluation in both women and men, women are more dissatisfied with their appearance and body (Hargreaves and Tiggemann, 2004; Frisén and Anneheden, 2014). Thinness, as a body ideal in the Western world, affects girls more than boys (Hargreaves and Tiggemann, 2004; McArthur et al., 2005; Golan et al., 2014). An additional cause is that *athletic participation* in adolescence is more common among boys and helps retain a positive body-image. In contrast, normal *physical changes in adolescent girls*, including increased body fat, go against Western cultural body ideals, whereas normal physical changes in boys, for example, muscle growth, are in line with such ideals (Rodgers et al., 2015).

Some studies on self-esteem change do not present gender differences (e.g., Trzesniewski and Donnellan, 2010; Twenge and Campbell, 2010). When gender data have been analyzed, other factors have sometimes overshadowed the results, such as the ethnicity of participants, which makes gender differences in self-esteem less discernible. Erol and Orth (2011) have found no significant gender moderation in their study of participants of mixed ethnicity from 14 to 30 years of age, but those authors admitted that ethnicity can be a confound.

The present study examines gender differences in five self-evaluation dimensions in two Swedish samples from 2013 and 1983 in the age-span 10–16 years and gender differences in secondary school (ages 16–18) from a 2013 sample. Gender differences are of special interest in Sweden – as it is recognized for striving toward gender equality.

### The Present Study and Societal Movements in Sweden During 1983–2013

Many equality reforms have been launched in Sweden since the 1980s, for example, goals for gender-equal representation in political and management positions and in recruitment to certain jobs, parental leave for men and women, and affordable, high-quality day care service that enables both parents to work full-time. The European Institute of Gender Equality (EIGE) gave Sweden the highest equality index, 83 out of 100, of all EU countries (EIGE, 2017). The question is whether the self-evaluation differences between girls and boys have evened out in Sweden. We examined two cross-sectional samples (from 1983 and 2013) in Sweden, to discover how gender differences in self-evaluation have changed from primary school to lower secondary school. We also had an additional group from secondary school from 2013 for which we examined gender differences.

A few other family and societal trends have taken place during the past 30 years in Sweden in addition to the equality trend. These trends may have had an influence on young people’s self-evaluations. One trend concerns altered child-rearing styles and parental habits. More children in the millennium generation than in the 1980s have been reared in a permissive way (Eberhard, 2014; Trifan et al., 2014). Millennium parents experience greater time pressure owing to the changed situation on the labor market in Sweden, with both parents working in nearly all families (Statistics Sweden, 2014). In order to compensate for their absence, parents may try to solve problems for their children instead of giving their children opportunities to learn to handle challenges. A positive effect of this permissiveness is more likely in the primary school group. However, early parental permissiveness may lead to poorer abilities to handle setbacks when challenges increase during adolescence (Spencer, 2008), something that in the older groups may influence self-evaluations negatively.

Other scholars have discussed the benefits of a less authoritarian upbringing (Trifan et al., 2014) because this method of child rearing means that fewer children lose their sense of agency, something that could lead to higher self-evaluation (Buri, 1989; Tafarodi et al., 2010; Trifan et al., 2014). Parents have 13 months of paid parental leave in Sweden, something that may have influenced parent-child relationships positively. *We explored whether there were differences in the total self-evaluation* scores for both comparison groups *in the 30-year comparison* (*H1explorative*). The total self-evaluation is the sum of the five self-evaluation dimensions.

There has been greater focus on sport activities in young children in recent decades, and this affects body evaluations. Today, many children practice several sports and during the primary school years, most children try a wide range of sport and hobby activities. Several studies describe a positive relation between sport participation and physical self-evaluation (Birkeland et al., 2012; Lindwall et al., 2014; Noordstar et al., 2016). *We assumed that there would be higher physical self-evaluation scores in primary school in 2013 than in 1983* (*H2a*).

Students in lower secondary school drop out of their sport activities (girls more often than boys), which might be a reason to believe that we will find a decrease in physical self-evaluation scores at least in girls in the older age group. Another reason might be the punishing body standards for girls that are even more pronounced nowadays and are spread via social media (Hargreaves and Tiggemann, 2004; Frisén et al., 2014; Golan et al., 2014). Less research suggests changes in boys’ physical self-evaluations. *Therefore, we explored whether there were generational differences in physical self-evaluation scores in 2013 in lower secondary school* (*H2b explorative*).

*We also assumed that there would be higher scores for psychological well-being self-evaluations for the primary school group in 2013* (*H3a*) along with the overall increase as reported internationally. A one-sided body ideal in lower secondary school would likely be a strong negative influence on psychological evaluation scores, particularly for girls. Reports on decreased psychological health in adolescent girls (Collishaw et al., 2010; Hagquist, 2010; Petersen et al., 2010; National Board of Health and Welfare, 2013; Fleming et al., 2014) might indicate a negative psychological self-evaluation trend mostly among girls. There is less evidence of generational trends in earlier research on boys’ psychological self-evaluations. *Therefore, we explored whether there were generational differences for psychological self-evaluation scores in lower secondary school in 2013 compared to 1983* (*H3b explorative*).

Another societal phenomenon that has changed in the past 30 years is that school practices have become less authoritative and more student centered (Enkvist, 2017), something that may boost student images of their school competence. Schools have also worked more actively with group dynamics in recent decades, for example, anti-bullying programs and self-esteem-raising activities. *We therefore assumed there would be higher school competence self-evaluation scores among the 2013 cohort than among the 1983 cohort for both age groups* (*H4*). Today’s generous 13-month parental leave and less authoritarian parenting (Trifan et al., 2014) might also have influenced family relations in a positive direction. *We assumed that there would be higher family related self-evaluation scores in 2013 than in 1983 for both age groups* (*H5*).

Another societal trend is that children already in their early years spend much more time with peers and in organized leisure-time activities with other adults than with their parents. Most children in Sweden also attend preschool between 1 and 5 years of age (84% in 2013 according to the National Agency for Education, 2018). Only about 25% of children did so in the 1970s (Bergqvist and Nyberg, 2001). This generation may, thus, be more familiar with spending time and functioning with other adults and peers and, therefore, *we assumed higher self-evaluation scores for relations to others in 2013 than in 1983 for both age groups* (*H6*).

We also explored gender differences in both age groups and the interaction between gender and generational differences. We found no Swedish research to support the formulation of a directed hypothesis for the primary school group. *Therefore, for the total score, and the five self-evaluation dimensions in primary school* (*ages 10–12*), *we explored whether girls and boys demonstrated differences* (*H7a explorative*). For the total score and the five self-evaluation dimensions in lower secondary school (ages 13–15), *we hypothesized that boys’ scores would be higher than girls’ scores in line with international studies* (*H7b*). We assumed that Swedish girls in lower secondary school (ages 13–15) would be exposed to and influenced by punishing Western body ideals to the same extent as other Western teenagers (Harter, 2012). For the same reason, *we also assumed that there would be higher total scores and higher scores in the five dimensions for male students than for female students in the secondary school sample from 2013* (*ages 16–18*) (*H7c*).

There is a lack of studies undertaken in the Nordic countries on generational change in self-evaluations. Gender and dimension-specific interactions have not been investigated in Sweden. The aim of the present study is, therefore, to investigate differences in five self-evaluation dimensions between two cross-sectional samples (1983 and 2013).

## Materials and Methods

### Participants

The participants in 2013 were 1303 children (759 girls, 544 boys) in the ages between 10 and 18 years from ethnically and socioeconomically mixed areas in southern Sweden. Most participants came from a small town (30,000 inhabitants) and a middle-sized town (300,000 inhabitants). The participants in 1983 were 2,662 students (1,331 girls, 1,331 boys) of the ages 10–15 years from the same areas as in 2013. Table 1 presents the number of participants for each school grade.

**TABLE 1:** Participants from each cross-sectional generation (data from 2013 and 1983).

**School grade**	**Girls**		**Boys**		**Total**	
	**1983**	**2013**	**1983**	**2013**	**1983**	**2013**
**Primary school, total**						
Grade 4	243	57	264	48	507	105
Grade 5	91	48	92	61	183	109
Grade 6	237	57	265	47	502	104
**Lower secondary school, total**						
Grade 7	352	97	320	85	672	182
Grade 8	95	84	88	56	183	140
Grade 9	313	108	302	111	615	219
**Secondary school, total**						
Grade 10	–	106	–	26	–	132
Grade 11	–	109	–	66	–	175
Grade 12	–	93	–	44	–	137
Total	1331	759	1331	544	2662	1303

The percentage of children with a foreign background was very similar in the two cross-sectional samples. 23 percent of the 1983 sample had two parents with a foreign background, and 22 percent stated that they spoke a language other than Swedish at home in 2013 (indicating ethnicity). Mainly two non-Swedish ethnic groups were represented in 1983: Finns and people from the former Yugoslavia, and no differences between the Swedish and non-Swedish groups were found (see Ouvinen-Birgerstam, 1984). Thirty-six languages were registered in 2013, of which the largest groups came from the Middle East and the Balkan States. No analyses are presented due to the small number of each ethnic group.

The participation rate was 91% in 1983, and it was 81.5% in 2013. The most common reasons for not participating were (1) absence on the day of testing and (2) not having brought the informed consent form to school. A passive parental consent procedure was employed for adolescents above 15 years of age, and active parental consent was used for children. Written information was sent to the students’ parents. Students could refrain from participation by talking to their respective teachers or by contacting the researchers directly. All participants gave informed consent.

All procedures performed were in accordance with the recommendations of the Swedish Central Review Board, which also approved the research study (the ethical reference number is 2010/475).

### Measures

Five-dimensional self-esteem was measured using ‘I think I am’ (ITIA; Ouvinen-Birgerstam, 1999). This test is frequently used in research in Sweden (Ouvinen-Birgerstam, 1999). The ITIA contains five dimensions: *physical self* (‘I’m good at sports’, ‘I don’t like my body’ Reversed item), *psychological well-being* (‘I am calm and controlled,’ ‘I often feel tense and nervous’ Reversed item), *school competence* (‘I am good at school,’ ‘I easily forget what I have learnt’ Reversed item) and two social self-dimensions: *family relations* (‘My parents are often pleased with me,’ ‘We fight a lot in my family’ Reversed item) and *relations to others* (‘I have a lot of friends,’ ‘I often feel lonely,’ Reversed item). The range of the total score is between −144 and 144 and for the dimensions the range is between −28 and 28 except for psychological well-being, which ranged between −32 and 32.

ITIA is designed for lower secondary school and secondary school students (ages 10–18). It consists of 72 items with four response alternatives: ‘agree completely,’ ‘agree partly,’ ‘disagree partly,’ and ‘disagree completely.’ Half of the items are formulated positively and half negatively.

The Cronbach’s alpha values for the 2013 sample were 0.79 for Physical self, 0.81 for Psychological well-being self, 0.75 for School Competence self, 0.84 for Family related self, 0.68 for Relations to others, and 0.92 for the total scale. Studies by the present research group have tested the convergent validity for the total ITIA self-evaluation score in relation to the Beck Youth Inventory and its 20-item general self-esteem subscale (Beck et al., 2004). Beck self-esteem showed a strong relation (*r* = 0.74) to the total ITIA scores (Leo and Persson, 2011). Forsman and Johnson’s (1996) basic self-esteem also had a strong relation (*r* = 0.81) to the total ITIA scores (Pybus, 2014). Basic self-esteem is a 13-item scale that measures emotionally based global self-esteem (Forsman and Johnson reported *r* = 0.79–0.85 in relation to Rosenberg’s self-esteem). These correlations indicate that the total ITIA score can be used to estimate global self-esteem. We present both the total score and the five dimensions in the results, despite the fact that some scholars advise against summing up different dimensions into a total score with their measures (Harter, 2012). ITIA’s five dimensions have since its construction (Ouvinen-Birgerstam, 1999) been used clinically to indicate different self-concept aspects, and the total score has been used as an estimate of global self-esteem. Cronbach alphas were not available for the data from 1983. The split-half method was used to evaluate the subscales’ reliability, indicating good reliability (Ouvinen-Birgerstam).

Inspection of histograms showed no clear deviations from normality for any dimensions except one, family related self-evaluation, for the 2013 sample. The skewness for most of the scales was clearly below a commonly used cut-point-range (i.e., between −1 and 1; Hair et al., 1998) ranging from −0.68 for the total score to −0.87 for self-evaluations for relations to others. For family-related self-evaluation the skewness was −1.4.

### Procedure

Data were collected from 31 schools in 2013 and from 27 schools in 1983. The test took about 30 min to complete. The dataset had some missing values, however, no item exceeded 1% missing values. The maximum of two items was replaced with the individual’s calculated mean for the remaining items for the corresponding subscales when the former was missing in some cases.

### Statistical Procedures

An online *t*-test calculator for two independent samples was used to investigate the self-evaluation differences between 1983 and 2013. We could not employ the Statistical Package for the Social Sciences (SPSS) in our comparison analyses because no individual raw scores were available for the students from 1983. The means, standard deviations, and numbers of participants from 1983, used in the present analyses are from the Ouvinen-Birgerstam report (1999). The formula for calculating an unbalanced factorial analysis of variance (ANOVA) suggested by Cohen (2002) was used to investigate the main effects and interactions of year (1983 and 2013) and gender on different dimensions of self-esteem. Independent *t*-tests were used to examine gender differences. Cohen’s *d* and $\eta_{\text{p}}^{2}$ were used to determine effect sizes. The following formula was used to calculate $\eta_{\text{p}}^{2}$ : $\eta_{\text{p}}^{2}\,=\frac{F\,\times d\,f_{\text{effect}}}{F\times d\,f_{\text{effect}}+\,d\,f_{\text{error}}\,}$ (Lakens, 2013). According to Cohen’s (1988) conventions, a small effect size is around *d* = 0.20, $\eta_{\text{p}}^{2}\,$ = 0.01; a medium effect size is around *d* = 0.50, $\eta_{\text{p}}^{2}\,$ = 0.06; and a large effect size is around or above *d* = 0.80, $\eta_{\text{p}}^{2}\,$ = 0.14.

A two-way multivariate analysis of variance (MANOVA) was conducted using SPSS version 20 (SPSS Inc., Chicago, IL, United States) to evaluate changes in self-evaluations across different grades and possible grade and gender interaction effects in 2013.

## Results

**FIGURE 1:**
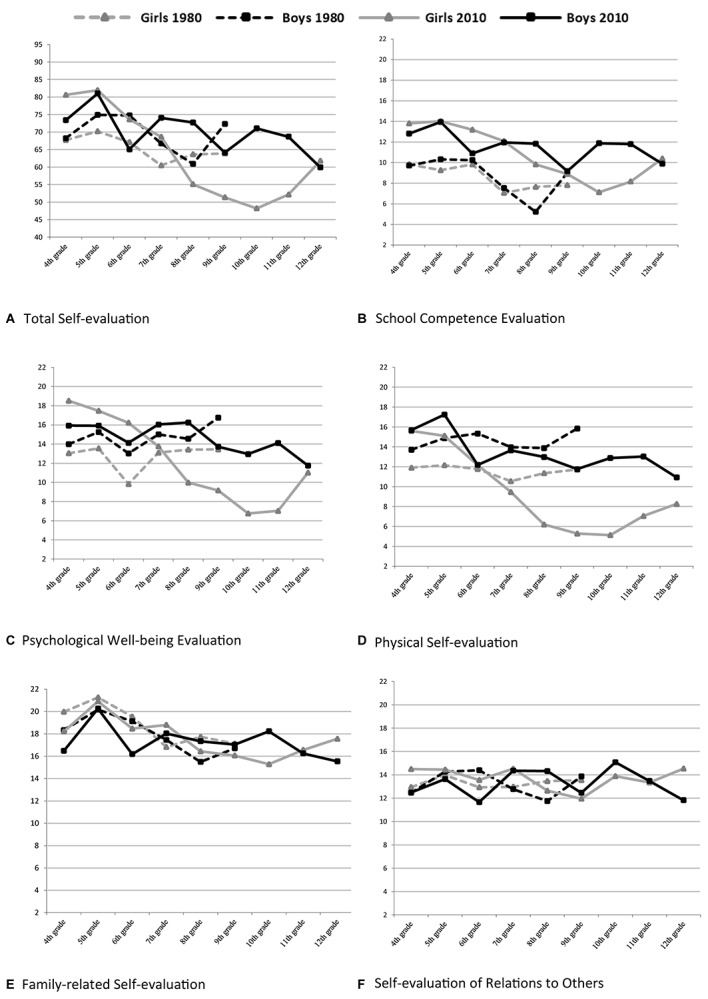
Means for total score and five self-evaluation dimensions in 2013 (ages 10–18) and in 1983 (ages 10–15).

The results from the two-way ANOVAs that examine the main effects of generation (1983 and 2013) and gender and a possible interaction of the two variables are presented separately for the overall sample, for primary school (ages 10–12) and for lower secondary school (ages 13–15). Only data from 2013 were collected for secondary school (ages 16–18), and gender and school grade differences are shown. Figure 1 presents the self-evaluation means for the total score and the five dimensions for grades 4–12 (ages 10–18) in 2013 and for grades 4–9 (ages 10–15 years) in 1983.^3^

### Generational and Gender Differences for the Overall Sample (Ages 10–15)

In response to *H1explorative* (i.e., an exploration of differences in total self-evaluation in 2013 compared to 1983) in the overall comparison (ages 10–15), a significant main effect of generation was found for the total self-evaluation. This effect occurred owing to higher self-evaluation scores for youth in 2013 than for youth in 1983, *F*(1,3517) = 25.72, *p* < 0.001, $\eta_{\text{p}}^{2}\,$ = 0.006. This indicates a very small effect size. There was also a significant main effect of gender in the total self-scores, to the advantage of boys, *F*(1,3517) = 11.35, *p* < 0.001, $\eta_{\text{p}}^{2}\,$ = 0.002. This also indicates a very small effect. The interaction effect was not significant. Table 2 summarizes the results.

**TABLE 2:** Mean (SD) self-evaluation scores and *F* ratios from ANOVAs to test main and interaction effects of generation and gender for total sample (ages 10–15).

**Variable**	**1983 Means (*sd*)**			**2013 Means (*sd*)**			**Main effect of generation**		**Main effect of gender**		**Interaction effect generation × gender**	
	**Girls**	**Boys**	**Total**	**Girls**	**Boys**	**Total**	***F***	***$\eta_{\text{p}}^{2}\,$***	***F***	**$\eta_{\text{p}}^{2}\,$**	***F***	**$\eta_{\text{p}}^{2}\,$**
Total	65.5	69.6	67.6	71.8	76.2	74.0	27.21***	0.006	12.01***	0.002	0.00ns	0.000
	(34.1)	(30.3)	(32.2)	(33.1)	(30.3)	(31.7)						

***p < 0.001; ns, non-significant.

### Generational and Gender Differences for Primary School (Ages 10–12)

Descriptive statistics and the results from the two-way ANOVAs for the primary school group are presented in Table 3.

**TABLE 3:** Mean (SD) self-evaluation scores and *F* ratios from ANOVAs to test main and interaction effects of generation and gender for primary school (ages 10–12).

	**1983 Means (*sd*)**			**2013 Means (*sd*)**			**Main effect of generation**		**Main effect of gender**		**Interaction effect generation × gender**	
	**Girls (*n* = 571)**	**Boys (*n* = 621)**	**All (*n* = 1192)**	**Girls (*n* = 162)**	**Boys (*n* = 154)**	**All (*n* = 316)**	***F***	**$\eta_{\text{p}}^{2}\,$**	***F***	**$\eta_{\text{p}}^{2}\,$**	***F***	**$\eta_{\text{p}}^{2}\,$**
Total	68.3 (35.4)	72.6 (31.0)	70.5 (33.2)	86.4 (27.0)	79.5 (29.0)	82.9 (28.0)	37.48***	0.024	0.41 ns	0.000	7.55**	0.005
Physical	12.0 (9.4)	14.6 (8.2)	13.3 (8.8)	16.1 (8.1)	16.8 (7.5)	16.4 (7.8)	33.32***	0.022	10.09**	0.007	3.22ns	0.002
Psychological well-being	13.2 (6.8)	15.0 (8.7)	14.1 (7.8)	17.4 (8.3)	16.0 (8.4)	16.7 (8.3)	26.25***	0.017	0.19 ns	0.000	9.92**	0.006
School competence	9.6 (9.3)	10.1 (8.8)	9.9 (9.0)	15.4 (6.7)	12.9 (7.0)	14.2 (6.9)	61.44***	0.039	3.46 ns	0.002	7.21**	0.005
Relations to family	20.3 (7.7)	19.2 (6.8)	19.7 (7.3)	21.8 (5.9)	19.6 (7.5)	20.7 (6.7)	4.46*	0.003	12.74***	0.008	1.56ns	0.001
Relations to others	13.3 (8.3)	13.7 (7.4)	13.5 (7.8)	15.8 (6.2)	14.1 (7.2)	14.9 (6.7)	9.26**	0.006	1.72ns	0.001	4.88*	0.003

***p < 0.001, **p < 0.01, *p < 0.05; ns, non-significant.

#### Total

In response to *H1explorative*, we found that primary school children in 2013 reported a significantly higher total self-evaluation score than their counterparts in 1983. There was no significant main effect of gender in the total self-evaluation, however, a significant interaction effect between generation and gender was found for the total score. Girls had higher self-evaluation scores than boys did in 2013, but not in 1983. Independent *t*-tests for generational gender differences showed that girls in 2013 reported significantly higher total self-evaluation scores than girls in 1983, *t*(731) = 5.98, *p* < 0.0001, *d* = 0.54. Boys in 2013 also reported a significantly higher total self-evaluation score than boys in 1983, *t*(773) = 2.64, *p* < 0.01, *d* = 0.22. Although boys reported higher scores for the total self-evaluation in 2013 than in 1983, a greater difference was found between girls in 2013 and girls in 1983.

#### Physical

In response to *H2a*, the results illustrate that primary school children’s physical self-evaluations were significantly higher in 2013 than in 1983 (see Table 3). There was also a significant main effect of gender to the advantage of boys in the physical dimension. No interaction effect was found. A higher difference was found for girls when generational gender differences were evaluated with independent *t*-tests, *t*(731) = 5.48, *p* < 0.0001, *d* = 0.45, than for boys, *t*(773) = 3.20, *d* = 0.27. This finding suggests that girls’ physical self-evaluation scores increased more between 1983 and 2013 than the scores for boys.

#### Psychological Well-Being

In response to *H3a*, the results showed that primary school children’s psychological self-evaluation scores in 2013 were significantly higher compared to 1983 (see Table 3). The main effect of gender was not significant, however, a significant interaction between generation and gender was found for this group, which indicates that girls’ scores had surpassed boys’ scores. Independent *t*-tests also showed that girls in 2013 reported significantly higher psychological self-esteem scores than girls in 1983, *t*(568) = 6.57, *p* < 0.0001, *d* = 0.58. No difference was found between 1983 and 2013 for boys, *t*(773) = 1.28, *p* = 0.20, *d* = 0.11.

#### School Competence

In response to *H4*, the results showed that primary school children’s competence self-evaluation scores in 2013 were significantly higher than in 1983. The main effect of gender was not significant, but a significant interaction effect was found between generation and gender, showing that boys reported slightly higher school competence scores in 1983, and girls reported higher scores in 2013 (see Table 3). Independent *t*-tests showed that girls in 2013 reported significantly higher school competence evaluation scores than girls in 1983, *t*(568) = 7.41, *p* < 0.0001, *d* = 0.66. Boys also reported significantly higher school competence evaluation scores in 2013 than in 1983, *t*(773) = 3,91, *p* < 0.0001, *d* = 0.33. Thus, again, a greater difference was found for girls although boys reported significantly higher school competence scores in 2013 than in 1983.

#### Family Relations

In response to *H5*, the results showed that primary school children’s family related self-evaluation scores in 2013 were significantly higher than in 1983, even though the effect size was very small (see Table 3). A main effect of gender was also significant in that girls scored higher than boys for both years. There was no interaction effect.

#### Relations to Others

In response to *H6*, the results showed that primary school children’s self-evaluations of relations to others in 2013 were significantly higher than in 1983, even though the effect size was very small (see Table 3). There was no significant main effect of gender, however, a significant interaction between generation and gender was found. Boys’ self-evaluations for relations to others were higher in 1983, whereas girls’ self-evaluations were higher in 2013.

#### Gender in Elementary School

In response to *H7a explorative* (i.e., an exploration of gender differences in self-evaluation in primary school students), two of five subscales showed the main effects for gender in the primary school group. One effect was to the advantage of girls but with a very small effect size; family related self-evaluation scores. One effect favored boys with a small effect; physical self-evaluations. Interestingly, there were interaction effects between generation and gender for the total score, psychological well-being, school competence, and relations to others evaluation scores, demonstrating that girls had higher scores in 2013 but boys had higher scores in 1983. These effect sizes were, however, very small (see Table 3).

### Generational and Gender Differences for Lower Secondary School (Ages 13–15)

Table 4 shows the descriptive statistics and results from the two-way ANOVASs for the adolescent group.

**TABLE 4:** Mean (SD) self-evaluation scores and *F* ratios from ANOVAs to test main and interaction effects of generation and gender for lower secondary school (ages 13–15).

	**1983 Means (sd)**			**2013 Means (sd)**			**Main effect of generation**		**Main effect of gender**		**Interaction effect generation × gender**	
	**Girls (*n* = 760)**	**Boys (*n* = 710)**	**All (*n* = 1470)**	**Girls (*n* = 289)**	**Boys (*n* = 252)**	**All (*n* = 541)**	***F***	**$\eta_{\text{p}}^{2}\,$**	***F***	**$\eta_{\text{p}}^{2}\,$**	***F***	**$\eta_{\text{p}}^{2}\,$**
Total	62.7 (32.9)	66.6 (29.5)	64.7 (31.2)	63.7 (32.3)	73.8 (30.7)	68.4 (32.5)	6.75**	0.003	20.00***	0.010	3.84*	0.002
Physical	11.2 (8.5)	14.6 (7.7)	12.9 (8.1)	9.6 (9.6)	15.0 (7.5)	12.3 (8.6)	1.97ns	0.001	110.30***	0.052	6.05*	0.003
Psychological well-being	13.3 (8.8)	15.5 (7.9)	14.4 (8.4)	11.9 (9.4)	16.2 (7.9)	14.0 (8.7)	0.61ns	0.000	56.00***	0.027	6.54*	0.003
School competence	7.5 (8.5)	7.2 (8.7)	7.4 (8.6)	10.9 (8.0)	10.9 (7.9)	10.9 (8.0)	68.88***	0.033	0.08ns	0.000	0.09ns	0.000
Relations to family	17.2 (9.7)	16.6 (7.9)	16.9 (8.8)	17.9 (8.8)	18.1 (7.5)	18.0 (8.1)	6.83**	0.003	0.28ns	0.000	1.06ns	0.000
Relations to others	13.3 (7.1)	12.8 (6.6)	13.1 (6.8)	13.5 (6.8)	14.5 (7.2)	14.0 (7.0)	7.41**	0.003	0.46ns	0.000	4.85*	0.002

***p < 0.001, **p < 0.01, *p < 0.05; ns, non-significant.

#### Total

In response to *H1explorative*, the results showed that lower secondary school students’ total self-evaluation scores in 2013 were significantly higher, however, with a very small effect size. No significant main effect of gender or significant interaction effect between generation and gender was found for the total score (see Table 4). Results from the independent *t*-tests analyses showed that boys’ total self-evaluation scores increased significantly from 1983 to 2013, *t*(960) = 3.30, *p* < 0.001, *d* = 0.24, while girls’ scores remained almost at the same level, *t*(1047) = 0.45, *p* = 0.66, *d* = 0.03. Gender differences for years 2013 and 1983 showed larger differences in 2013 for the total score, *t*(540) = 3.72, *p* = 0.0002, *d* = 0.32 than for gender differences in 1983, *t*(1468) = 2.38, *p* = 0.02, *d* = 0.12.

#### Physical

In response to *H2b explorative* (i.e., exploration of generational differences for physical self-evaluation in 2013 compared to 1983, in the lower secondary school group), results from the independent *t*-tests demonstrated significant gender differences indicating that boys’ scores increased slightly (though non-significantly) between 1983 and 2013, *t*(960) = 0.72, *p* = 0.47, *d* = 0.06, whereas girls’ physical self-evaluation scores decreased significantly, *t*(1047) = −2.61, *p* = 0.005, *d* = −0.18.

In response to *H7b* (i.e., an assumption of higher scores for boys’ physical self-evaluation than for girls), the results showed a significant main effect of gender, to the advantage of boys in both generations. An interaction effect between generation and gender was also significant, even though only a very small effect size was found (see Table 4), indicating that the gender gap had increased between 1983 and 2013. Results from the *t*-tests showed that a larger gender difference existed for physical self-evaluation scores in the 2013 sample, *t*(539) = −7.33, *p* < 0.001, *d* = 0.62 than in the 1983 sample, *t*(1468) = 8.05, *p* < 0.001, *d* = 0.42.

#### Psychological Well-Being

In response to *H3b explorative* (i.e., exploration of generational differences in psychological self-evaluation scores in 2013 compared to 1983, in the secondary school group), the results from independent *t*-test analyses showed that boys’ scores had increased slightly compared to 1983, *t*(960) = 1.21, *p* = 0.22, *d* = 0.10, while girls’ psychological self-evaluation scores had decreased significantly in 2013, compared to 1983, *t*(1047) = −2.39, *p* = 0.009, *d* = −0.16.

In response to *H7b* (i.e., an assumption of higher scores for boys’ psychological well-being self-evaluation than for girls), the results showed that the main effect of gender was significant, to the advantage of boys in both years (see Table 4). The interaction effect between generation and gender was also significant, indicating a greater gender gap in 2013. However, the effect size was very low. Results from the *t*-tests showed a larger gender difference for psychological self-evaluation scores in 2013, *t*(540) = 5.46, *p* = 0.0001, *d* = 0.47, compared to 1983, *t*(1468) = 4.77, *p* = 0.0001, *d* = 0.25.

#### School Competence

In response to *H4*, the results demonstrated that lower secondary school students’ competence self-evaluation scores in 2013 showed a significant increase compared to 1983 (see Table 4). There were neither significant main effects of gender nor significant interaction effects.

#### Family Relations

In response to *H5*, the results showed that lower secondary school students’ scores for family related self-evaluation in 2013 were significantly higher than in 1983, although the effect size was very small (see Table 4). There were neither significant main effects of gender nor significant interaction effects.

#### Relations to Others

In response to *H6*, the results showed that lower secondary school students’ scores for relations to others in 2013 were significantly higher than in 1983, although the effect size was very small (see Table 4). There were neither significant main effects of gender nor significant interaction effects.

#### Gender in Lower Secondary School

Summarizing the results of *H7b* (i.e., an assumption of higher scores for lower secondary school boys than for girls for the total self-evaluation and the five dimensions), the hypothesis was partly supported. A main effect for gender was found in the total score and two of the dimensions: physical and psychological well-being (with effect sizes between low to medium, see Table 4).

### Gender Differences in Secondary School in 2013 (Ages 16–18)

Data for secondary school students were only collected in 2013, and only gender differences were calculated as a result. Table 5 presents a summary of the results.

**TABLE 5:** Mean (SD) self-evaluation scores for secondary school students (ages 16–18).

**Variable**	**2013 Means (*sd*)**		***F***	**$\eta_{\text{p}}^{2}\,$**
	**Girls (*n* = 308)**	**Boys (*n* = 136)**		
Total	53.7 (30.0)	66.3 (30.5)	15.78***	0.15
Physical	6.8 (8.6)	12.3 (8.1)	40.56***	0.08
Psychological well-being	8.1 (9.7)	13.1 (9.3)	25.49***	0.06
School competence	8.5 (7.3)	11.2 (7.4)	13.09***	0.03
Relations to family	16.4 (8.6)	16.4 (7.3)	0.00 ns	0.00
Relations to others	13.9 (5.5)	13.2 (6.1)	1.19 ns	0.00

***p < 0.0001; ns = non-significant.

In response to *H7c* (i.e., an assumption of higher self-evaluation scores for male compared to female secondary school students, in total self-evaluation and the five dimensions), a one-way MANOVA revealed a significant multivariate main effect for gender, Wilks’ λ = 0.847, *F*(5,438) = 15.78, *p* < 0.0001, $\eta_{\text{p}}^{2}$ = 0.15, indicating that male students (*M* = 66.3, *SD* = 30.5) reported significantly higher total self-evaluation scores than female students (*M* = 53.7, *SD* = 30.0). The gender differences for the first 2 years of secondary school were of high or medium effect (*d* = 0.72 first year and *d* = 0.54 second year), but disappeared the third year of secondary school.

Significant univariate main effects for gender indicate that male students reported significantly higher physical self-evaluation, *F*(1,442) = 40.56, *p* < 0.0001, $\eta_{\text{p}}^{2}$ = 0.08, psychological well-being self-evaluation, *F*(1,442) = 25.49, *p* < 0.0001, $\eta_{\text{p}}^{2}$ = 0.06, and school competence evaluation, *F*(1,442) = 13.09, *p* < 0.0001, $\eta_{\text{p}}^{2}$ = 0.03, than female students (see Table 5). As no gender differences were found for the two relational self-evaluation scales in secondary school, *H7c* was thus supported in three of the five dimensions, apart from the total score, which demonstrated significant gender differences.

### Self-Evaluation From Fourth to Twelfth Grade in 2013 (Ages 10–18)

A two-way MANOVA was employed to explore changes in self-evaluation scores across different grades and possible grade and gender interaction effects in 2013. Univariate main effects for grade and gender and interaction effect for grade × gender were also evaluated and are presented in Table 6.

**TABLE 6:** ANOVAs to test effects of grades 4 to 12 (ages 10–18) and gender on self-evaluation.

	**Main effect of grade**		**Main effect of gender**		**Interaction effect grade × gender**	
	***F***	**$\eta_{\text{p}}^{2}\,$**	***F***	**$\eta_{\text{p}}^{2}\,$**	***F***	**$\eta_{\text{p}}^{2}\,$**
Total	5.07***	0.030	23.73***	0.09	1.86**	0.012
Physical	14.36***	0.082	67.96***	0.050	3.72***	0.023
Psychological well-being	11.80***	0.069	20.10***	0.015	6.00***	0.036
School competence	8.01***	0.048	3.20 ns	0.002	3.02**	0.018
Relations to family	3.59***	0.022	0.49 ns	0.000	1.27 ns	0.008
Relations to others	2.43*	0.015	1.53 ns	0.001	1.83 ns	0.011

***p < 0.0001, **p < 0.001, *p < 0.01; ns, non-significant.

A two-way MANOVA revealed a significant multivariate main effect for gender, Wilks’ λ = 0.915, *F*(5,1280) = 23.73, *p* < 0.0001, $\eta_{\text{p}}^{2}$ = 0.09, a significant multivariate main effect for grade, Wilks’ λ = 0.856, *F*(40,5582,19) = 5.07, *p* < 0.0001, $\eta_{\text{p}}^{2}$ = 0.03, and a significant gender × grade interaction, Wilks’ λ = 0.944, *F*(40,5582,19) = 1.86, *p* < 0.0001, $\eta_{\text{p}}^{2}$$\eta_{\text{p}}^{2}$ = 0.012 (Table 6).

Univariate interaction effects were examined given the significance of the overall tests. The results indicate a significant gender × grade interaction for the dimensions physical self-evaluations, psychological well-being, and school competence (see Table 6 for details).

#### Physical

Girls reported significantly lower scores in grade 7, *t*(181) = −3.08, *p* = 0.002, *d* = −0.47 for physical self-evaluation, which was almost at the same level for girls and boys in grade 6 (girls: *M* = 13.6, *SD* = 8.9; boys: *M* = 0.13.9, *SD* = 8.7). This continued to decrease until grade 9, with the largest gender differences found in grade 10, *t*(130) = −3.85, *p* = 0.001, *d* = −0.87. Boys’ scores fluctuated somewhat from grade to grade with significant decreases (as compared to the scores in the lower grades) in grades 6, 9, and 12 (see Figure 1).

#### Psychological Well-Being

Similar trends regarding girls’ and boys’ developmental paths were found for psychological self-evaluation scores. The greatest gender difference for psychological well-being self-evaluation was found in grade 8 at the age of 14 [*t*(138) = −4.11, *p* = 0.0001, *d* = −0.69], indicating that boys’ scores significantly exceeded girls’ scores.

#### School Competence

The greatest gender difference in school competence evaluations was found in grade 10 at the age of 16 [*t*(130) = −2.65, *p* < 0.009, *d* = −0.60], showing that boys evaluated their school competence higher than girls did.

## Discussion

The results showed that there was an overall generational increase in self-evaluations between 1983 and 2013 (*H1explorative*), though the effect size was very small.

School level was an important factor that differentiated between a substantial increase in primary school ($\eta_{\text{p}}^{2}$ = 0.025) in 2013 and very small (but significant) differences in lower secondary school ($\eta_{\text{p}}^{2}$ = 0.003) in the comparison of self-evaluation scores between the cross-sectional samples. large cohort differences among younger children (*d* = 0.55 in Twenge and Campbell, 2001 between 1980 and 1993) were also found in an American meta-study. The effect size of that study was large also for lower secondary school (*d* = 0.67) on a self-evaluation scale. Other studies with data collected closer in time to our sample and focused on global self-esteem found small changes in adolescence. Gentile et al. (2010) have demonstrated a small effect size between 1988 and 2004 for secondary school (*d* = 0.39), and only a very small effect size when controlling for ethnicity (*d* = 0.17). Twenge and Campbell (2010) found a very small change among 8th graders between 1988 and 2005 (*d* = 0.12). Other researchers have found no cohort changes for global self-esteem scores during the lower secondary school years (Bachman et al., 2011, between 1993 and 2008).

### Primary School Changes (Ages 10–12)

An increase in self-evaluation scores (the total score plus all dimensions) was found for the 2013 sample compared to the 1983 sample in *primary school.* We suggest that increases in primary school children’s total and family related self-evaluation scores might be an effect of changed parenting. Trifan et al. (2014) found that since the 1980’s parenting in Sweden has become less authoritarian, something that together with extended parental leave for both parents may have affected levels of total self-evaluation scores and family related self-evaluation. More relative influence and attention early in life may have boosted children’s self-evaluation scores.

An interaction between generation and gender was found to the advantage of primary school girls in the total score, psychological well-being, school competence, and relations to others dimensions (though very small effect sizes). Primary school boys had higher scores for physical self-evaluation, and girls had higher scores for family relations both years. Thus, since the 1980s, girls have surpassed boys in their scores for all but one self-evaluation dimension in primary school. The gender trend was the opposite in Twenge and Campbell’s American meta-analytic self-evaluation study from 2001 (boys scored higher), and this result is also in line with what American self-esteem studies have generally found (see meta-study by Kling et al., 1999). In one study, however, gender differences were found in a participant group of Americans of Mexican origin in global self-esteem, which favored girls in lower secondary school (*d* = 0.21) (Orth et al., 2014b). In a meta-analysis of dimensional self-evaluation (predominantly US samples), boys exceeded girls on four of the investigated ten dimensions, but girls scored higher in behavioral conduct and moral self-evaluation (Gentile et al., 2009). The other dimensions were equal.

In the physical dimension and regarding *H2a*, girls’ self-evaluation scores had risen with a medium effect size (*d* = 0.51), while boys’ scores increased with a small effect (*d* = 0.29). Perhaps the greater focus on sport activities for young children in recent years can explain the boost in physical self-evaluation scores. Children often practice several sports during primary school years in Sweden. Boys had higher scores (only) in the physical dimension in the primary school group, and this is interesting to note because this was the dimension that deviated the most in the adolescent groups (in the same direction). Other international studies have revealed substantially lower physical self-evaluation scores for girls in lower secondary school than for boys. In a meta-analysis, Gentile et al. (2009) demonstrated a small effect size (*d* = 0.30) for gender differences on the appearance dimension for 5- to 10-year-old children. Frisén and Anneheden (2014) found similar results in a Swedish sample (*d* = 0.24) of 10-year-olds’ appearance scores. The physical subscale used in the present study was a mixture of questions concerning appearance and body-related self-evaluations, so the comparisons do not match perfectly. Psychological well-being evaluation scores were higher for the sample from 2013 than for the sample from 1983, which supports *H3a*. Primary school girls’ scores increased in the psychological well-being dimension whereas boys’ scores increased very little between the 2 years ($\eta_{\text{p}}^{2}$ = 0.006 indicating a very small effect size for Generation × Gender interaction).

We suggest that the high self-evaluation scores (total score and four of the dimensions) for primary school girls in Sweden may be a culture-specific phenomenon. Gender equality issues in Sweden have found their way into day care and primary school practice. All children are encouraged to try all types of activities in contemporary day care centers. Most day care centers lack gender-stereotyped rooms, such as doll rooms or woodwork rooms. This early training may have an effect on primary school girls’ total and psychological well-being self-evaluation scores. Swedish primary school girls’ competence advantage (reported in national tests, see National Agency for Education, 2013) can more easily be translated into overall self-evaluation advantages.

The greatest generational change effects in specific dimensions were found in school competence evaluation and supported *H4*. Significant differences were found in school competence self-evaluation scores for both age groups ($\eta_{\text{p}}^{2}$ = 0.04 for primary school and 0.03 for lower secondary school). This indicates a general trend in Sweden toward more positive school competence evaluation scores. Terracciano et al. (2005) have also found that younger generations scored higher on competence evaluations in a US cohort comparison. An increase may have positive and negative implications. On the one hand, the relation between beliefs about competence and actual performance is often positive, for example, high self-efficacy beliefs are often linked to good performance (Bandura, 1997). However, the relation to actual knowledge was not investigated in the present study. On the other hand, there are alarming reports about falling school results among Swedish students in international comparisons (e.g., PISA, see National Agency for Education, 2013) that can be compared with the present study’s increase in school competence evaluation scores. It has been suggested that students today do not get appropriate feedback on their knowledge level in school. Some researchers assert that changed school practices might result in inflated self-esteem (Gentile et al., 2010). Grade inflation can explain some of the rise in school competence evaluation scores in lower secondary school and secondary school groups (Vlachos, 2010). However, this explanation is not valid for primary school because Swedish primary students are not graded. More research is needed to investigate whether students’ school competence evaluation scores are inflated or realistic and how different types of feedback influence competence self-evaluation scores.

Slightly higher scores for relations to others (2013 as compared with 1983) were found in the present study and supported *H6* (but with a small effect size). This can partly be explained by Sweden’s high rate of day care attendance during the early years (National Agency for Education, 2018) and that children spend a considerable amount of time in after-school programs and other organized leisure-time activities during primary school years. The 2013 generation might, therefore, be more familiar with spending time and functioning with adults and peers outside the family. Research supports the link between social ability and day care attendance (if day care is of reasonable quality; e.g., Andersson, 1992).

### Lower Secondary School Changes (Ages 13–15)

The picture looks different in lower secondary school. The total self-evaluation scores had hardly risen for girls in a comparison of 1983 and 2013, but the self-evaluation scores for boys had increased significantly. Girls’ scores in the 2013 sample had decreased in comparison to 1983 for the physical and psychological well-being dimensions. Wångby et al. (2005) similarly found an increase in self-esteem problems for 15-year-old Swedish girls in a comparison between 1976 and 1996, and the effect size (*d* = 0.16) was similar to the decrease in physical and psychological well-being scores in the present study (*d* = 0.18 and 0.17, respectively). The opposite trends with generational decreases in self-evaluation scores for lower secondary school girls and increases for primary school girls might partly be an effect of more developmental challenges in the lower secondary school group. It is possible that more demands for responsibility are laid on the shoulders of female youth early on. Another explanation for the dip in girls’ self scores is that appearance perceptions drive other self-evaluations, including global self-esteem (Harter, 2012). Lower secondary school girls’ self-evaluation scores are particularly affected because of an increased body dissatisfaction in early adolescence as a consequence of the natural body development of females (fat increase), which in turn moves girls further away from Western body ideals (Westerberg-Jacobson et al., 2012; Rodgers et al., 2015).

One question remains however: Why does the adolescent dip go deeper in 2013 than in 1983 for girls? One oft-inferred explanation is that body image ideals in media have become increasingly unrealistic over time (Hargreaves and Tiggemann, 2004; Frisén et al., 2014; Golan et al., 2014). Why do not boys’ mean scores demonstrate any clear-cut deflation in self-evaluation scores? It is possible that society’s pressure on young men increases later on at ages not covered by the present study (indicated by a tendency toward a decrease in scores of the oldest secondary school male participants). Adolescent boys’ natural body development, muscle growth, is in line with Western body ideals, something that might protect them from body dissatisfaction during their adolescent years. Hargreaves and Tiggemann (2004) have compared the experimental effects of girls’ and boys’ exposure to ideal body images in television commercials and found increased body dissatisfaction in girls but not in boys. They have argued that media portrayals of a thinness ideal are more prevalent than portrayals of a muscular ideal (which was used when testing boys in their study) and this may help explain their results. Hargreaves and Tiggemann have suggested that girls might process appearance information more deeply and more automatically owing to the higher prevalence of female beauty ideals. However, appearance investment as a personality trait also predicts a more negative effect of the thinness and muscular ideals on girls and boys. Men are, thus, not fully protected. Some researchers have argued that men will become more vulnerable in the future because the muscular ideal is becoming increasingly pervasive in commercials and fashion magazines (Leit and Pope, 2001). Research has also demonstrated that men develop vulnerability to ideal media images somewhat later in life, in young adulthood. However, these are unattainable muscle ideals instead of thinness (Leit et al., 2002; Räty et al., 2005; Frisén et al., 2015).

Finally, participation in sports activities decreases during adolescence, which may be another reason for girls’ dip in physical self-evaluation scores, as noted in the present study (both years) and in international ones (e.g., Kling et al., 1999). Studies report a positive relation between physical self-esteem and physical activity (Birkeland et al., 2012; Lindwall et al., 2014; Noordstar et al., 2016). Perhaps girls would be less vulnerable to objectification if they maintained their sport activities.

School competence evaluation scores for primary school rose for both girls and boys, and no gender difference existed in either generation in lower secondary school.

The relational self-evaluation scores showed a small increase for adolescent girls (also for boys), possibly indicating that most of them feel they have supportive relations despite psychological and body-related self-concept problems for girls.

The gender gap in lower secondary school had increased in 2013 compared to 1983 for total, physical, and psychological well-being evaluation scores (to the advantage of boys). The gender difference in 2013 was greater for the physical (*d* = 0.62) and psychological well-being (*d* = 0.47) dimensions than in 1983 (physical, *d* = 0.42; psychological, *d* = 0.25). These results are in accordance with what generational meta-analyses of physical self-evaluation scores have shown in predominantly US samples (Feingold and Mazella, 1998; Gentile et al., 2009).

### Grade and Gender Differences in Secondary School (Ages 10–18) in 2013

The grade-by-grade comparison for the three secondary school years in 2013 showed significant differences across grades and gender. Girls generally reported lower self-evaluation scores than boys did for the total score, partly confirming *H7c*. The gender difference in the two first years of secondary school was of medium effect size (*d* = 0.72 and 0.54, respectively). However, the difference had disappeared in the total score from the final year of secondary school. Boys also had higher scores for three of the five dimensions: physical, psychological well-being, and school competence self-evaluation. Higher scores among boys in secondary school have been confirmed by several other studies. Räty et al. (2005) have found gender differences with medium effect size (*d* = 0.51) for Swedish 18- to 20-year-olds using the same self-evaluation measure used in present study. The meta-study by Gentile et al. (2009) found small gender differences for 14–17-year-olds (*d* = 0.30 for appearance and 0.36 for self-satisfaction).

The gender difference in Sweden was even greater in secondary school (ages 16–18) than in lower secondary high (ages 13–15). It is interesting to consider why gender equality practice and policies seem to be able to influence self-evaluations in primary school but not during adolescence. Are secondary schools more traditional in their gender expectations? Earlier research has found that school climate influences self-concept (Morin et al., 2013), and perhaps the influence is different for female and male students. The transition into a new school situation, which has been demonstrated to entail more social comparison and competition (Eccles et al., 1993), may have varying impacts on different students.

Boys’ scores demonstrated no clear-cut dip during the age span covered in the present study. Nevertheless, a late adolescence decrease for boys might possibly be detectable. While girls’ scores rose during the last year of data collection, boys’ scores fell slightly (see Figure 1). In line with these results, Räty et al. (2005) have found that Swedish 21-year-old girls surpassed their male peers on self-evaluation measured with the same instrument as in the present study. However, their sample was too small for grade 12 (*N* = 47) to draw definite conclusions – as was the present sample secondary school, grade 12 (*N* = 137). Similarly, Frisén et al. (2015) have indicated that a late negative trend in body self-concept existed for men in their early twenties, however, men still scored higher than women on this issue. Others have found a constant global self-esteem increase from the age of 15 for both girls and boys (Erol and Orth, 2011; von Soest et al., 2016). The gender gap in a Norwegian study continued to decrease in young adulthood, with women and men reaching an equal level of self-esteem at the age of 31 (von Soest et al., 2016). There is a need for further studies in Sweden to ascertain whether women’s self-esteem also becomes equivalent to men’s self-esteem during young adulthood.

### Implications of the Results

The question arises whether there is a ‘knowledge – belief’ gap in Sweden among young people, meaning that they have inflated beliefs about their knowledge levels. Research must investigate whether there is such a gap, and if so, actions should be taken to re-establish a reasonable relation between school competence evaluations and actual knowledge levels. If pedagogical failure or grade inflation underlies this self-evaluation increase, teachers should avoid exaggerated praise/grades and teach students to make realistic self-evaluations.

Perhaps communities should not arrange general self-esteem-boosting programs for students, but rather tailor such programs to groups identified as having (unwarranted) low self-esteem (Morin et al., 2011, 2013; Birkeland et al., 2012). Based on the present results, in which most groups demonstrated increases in self-evaluation scores, one could infer that such programs should be directed toward certain groups of teenage girls and predominantly concern the physical and psychological well-being self-dimensions.

### Limitations

The present study has a number of limitations. One is that only two sampling points (1983 and 2013) were compared. Preferably, more measure points should have been included as well as measures for societal trends. The time periods covered in different studies influence the results, which is why it is difficult to make comparisons with other studies. Park et al. (2014) have found that economic crises, among other things, affect self-esteem trends in that self-esteem decreases during recessions. Many societal and economic trends may have influenced students’ self-evaluation scores during the studied period, and those mentioned in the discussion in Sections “Primary School Changes (Ages 10–12),” “Lower Secondary School Changes (Ages 13–15),” “Grade and Gender Differences in Secondary School (Ages 10–18) in 2013,” and “Implications of the Results” were not analyzed statistically.

Another limitation is that socio-economic variables and separate school data were not available. The 2013 sample was collected in the same region and with the same mixture between schools from small and middle-sized towns as in 1983, but not all schools were the same. The sample from 2013 was smaller, which might make it less representative. Had we had a larger sample from 2013, we could have made more sub-group comparisons, for example of self-evaluation differences between ethnic groups, SES and schools. Earlier studies have only found minor SES-influence on self-measures (e.g., Twenge and Campbell, 2002) and research on school climate has shown effects on self-evaluations (Morin et al., 2013). In future studies, the SES-influence particularly on the dimension school competence evaluation should be investigated.

Other factors vary between the international studies we compared with, for example, instrument use. We included comparisons with self-esteem studies, even though the present study used a dimensional self-evaluation scale, and some authors stress that these constructs are different (Marsh et al., 2006; Harter, 2012). However, as discussed in the methods section, Section “Measures” (Leo and Persson, 2011; Pybus, 2014), we found a substantial relation between the total self-evaluation score and self-esteem measures, so comparisons may still be relevant.

The measurement can be further developed in the future to take into account later findings of more specific self-evaluation dimensions (Marsh et al., 2006; Harter, 2012), which the present measurement does not mirror. The physical scale of the Swedish measurement combines appearance with athletic physical evaluations, which other scholars have found to be two separate dimensions. Another is the combination of friends and teachers into a generic ‘relations to others dimension’ that could be divided into two dimensions. The present measure is, however, a frequently used instrument in Sweden. Finally, our analyses of the data distributions were limited since we did not have access to raw data from 1983. However, normality analyses for the 2013 sample were performed, showing no clear deviations from normality for any of the dimensions except family related self-evaluation.

## Conclusion

Generational change in five self-evaluation dimensions and total score was found in a comparison between two Swedish cross-sectional samples collected 30 years apart. The increase in self-evaluation scores for primary school girls was higher than for primary school boys in 2013 compared to 1983. The drop in lower secondary school girls’ self-evaluation scores (physical and psychological well-being and total score) was deeper in 2013 than in 1983. Lower secondary school boys did not show any change. A general trend toward an increase over time was discerned in one single dimension – school competence self-evaluation. Young Swedes in both age groups in 2013 rated their school competence higher than young Swedes in 1983.

In the more detailed analyses of the 2013 sample, the finding of a grade by gender interaction for the physical, psychological well-being dimensions and the total score is noteworthy. Girls in primary school began reporting higher self-evaluation scores in Sweden in 2013 than boys, and then there was a turning point somewhere around the age of 12 after which girls’ scores decreased until the age of 15. The girls’ scores did not recover until the age of 18, by which time the girls’ curve rose and even surpassed boys’ self-evaluation scores in a few of the dimensions.

Possible explanations for generational differences can be found in different societal trends, such as changed child-rearing styles, altered leisure-time habits, social media usage, and educational practices.

## Data Availability Statement

The raw data supporting the conclusions of this article will be made available by the authors, without undue reservation, to any qualified researcher.

## Ethics Statement

All procedures performed were in accordance with the recommendations of the Swedish Central Review Board, which also reviewed and approved the research study as human participants were involved (the ethical application reference number is 2010/475). Written informed consent to participate in this study was provided by the participants’ legal guardian/next of kin.

## Author Contributions

EH and DD contributed to the conception and design of the work; the acquisition, analysis, and interpretation of data for the work; and the drafting and revising of the work. PB contributed to the conception and design of the work and the revising of the work.

## Conflict of Interest

The authors declare that the research was conducted in the absence of any commercial or financial relationships that could be construed as a potential conflict of interest.

## Footnotes

1 This generation is sometimes also referred to as ‘generation me’ or ‘generation Y,’ born between 1985 and 2004.

2 This generation was born between 1965 and 1984.

3 In the study, primary school refers to students between the ages of 10 and 12 attending ‘mellanstadiet’ in Sweden (grades 4–6), the term lower secondary school is equivalent to ‘hogstadiet’ with students between the ages of 13 and 15 (grades 7–9), and finally secondary school refers to Swedish ‘gymnasiet’ for students between the ages of 16 and 18 (grades 10–12).
